# A Multilevel Spatial Model to Investigate Voting Behaviour in the 2019 UK General Election

**DOI:** 10.1007/s12061-023-09563-6

**Published:** 2024-01-11

**Authors:** Kevin Horan, Chris Brunsdon, Katarina Domijan

**Affiliations:** 1 Hamilton Institute, Maynooth University, Maynooth, Ireland; 2 National Centre for Geocomputation, Maynooth University, Maynooth, Ireland; 3 Deparment of Mathematics and Statistics, Maynooth University, Maynooth, Ireland

**Keywords:** Spatial regression, Hierarchical model, Intrinsic conditional autoregressive models, UK general election 2019, Butler swing

## Abstract

This paper presents a modelling framework which can detect the simultaneous presence of two different types of spatial process. The first is the variation from a global mean resulting from a geographical unit’s ‘*vertical*’ position within a nested hierarchical structure such as the county and region where it is situated. The second is the variation at the smaller scale of individual units due to the ‘*horizontal*’ influence of nearby locations. The former is captured using a multi-level modelling structure while the latter is accounted for by an autoregressive component at the lowest level of the hierarchy. Such a model not only estimates spatially-varying parameters according to geographical scale, but also the relative contribution of each process to the overall spatial variation. As a demonstration, the study considers the association of a selection of socio-economic attributes with voting behaviour in the 2019 UK general election. It finds evidence of the presence of both types of spatial effects, and describes how they suggest different associations between census profile and voting behaviour in different parts of England and Wales.

## Introduction

While it is common to capture spatially varying phenomena using models based on a multi-level framework or using an autocorrelation component, the objective of this study is to build a model to test for the simultaneous presence of both of these processes. This is done by combining a nested tree-structure of administrative boundaries with an additional spatially autocorrelated process at the lowest level of the geographical hierarchy. This framework makes it possible to allocate spatial variation according to process and geographical scale. The output of such a model is a set of spatially-varying coefficients at different hierarchical levels, a spatially autocorrelated random component at the lowest level of the hierarchy, and an estimation of the relative contribution of each to overall variance. The autocorrelated component can take the form of a collection of random intercepts, random coefficients, or both, according to specification.

It thus falls into the category of hierarchical spatial autoregressive modelling (HSAM) introduced by Dong and Harris (2015), except that it uses a frequentist rather than a Bayesian approach.

The model is applied to a case study of voter behaviour, examining the association of census variables which have been used by Beecham et al. (2018) to study spatial variation in recent voting patterns, with voters’ tendency to change allegiance from Labour to the Conservative Party in the 2019 UK general election. By combining hierarchical and autocorrelated processes, it finds that at different levels of geographical hierarchy, the association of these variables with voting outcomes varies in magnitude and direction across the study area. A constituency with a greater ethnic diversity, for example, has markedly different associations with voting behaviour in London and parts of the East Midlands than it does in the North East. It also finds evidence of spatial effects reflecting a similarity among neighbouring locations, which are free to operate across administrative boundaries. Certain places have an additional tendency to vote a certain way which cannot be explained by census profile or nested location. Overall, it estimates that approximately 27% of the variation between constituencies is accounted for by the spatial hierarchy, while a further 41% can be attributed to constituency level ‘*spillover effects*’ from adjoining constituencies.

This multilevel spatial approach to analysis is suitable not only for the study of elections, but can be easily adapted to any context or distributional family where a combination of spatial processes are hypothesised to be at play simultaneously, such as disease mapping (Vranckx et al., 2019) and survival models for businesses (Bivand & Gómez-Rubio, 2021).

### Spatial Processes

The incorporation of spatial processes into models is a generic issue across quantitative human geography (Fotheringham & Brunsdon, 1999). Firstly, human geography is not like physical geography in that it does not necessarily obey universal laws. While it may be possible to identify associations of certain covariates with an outcome, it is not always the case that these associations will be the same in different places. Furthermore, the observations within a dataset which has a geographic component can not be seen as independent. As Tobler’s First Law of Geography (Tobler, 1970) states, “everything is related to everything else, but near things are more related than distant things”. These additional realities should be reflected in the structure of a spatial model.

#### Hierarchical Process

One way to account for location is by using hierarchical or multi-level models, which model different spatial units at different levels. Much of the work done by Goldstein (1987) in the development of multi-level models was focused on education research. In such a context, pupil outcomes could be seen as depending not only on the various decisions of local education boards (highest level), but within each of those, on the policies of different head-teachers in each school, and subsequently on the skills of or decisions made by each teacher within each school. The lowest level would then be the individual pupil. These different levels, however, could also be nested geographical divisions, such as regions, counties and electoral constituencies. The introduction of geographical levels was developed by Jones (1991), and such a framework has previously been applied to voting behaviour (Jones et al., 1998). This nested process can be characterised as ‘*vertical*’ in the sense of correlations extending up and down through a branching tree.

This structure, however, requires us to know a priori what the appropriate scales are and to introduce hard boundaries accordingly. Standard mixed modelling assumes that beyond the random effects at these scales, no further correlation exists. In a geographical context, while this may explain a certain amount of the process, there could be a further spatial process which is better described using a spatially-autocorrelated framework.

#### Spatial Autocorrelation Process

Spatial processes can manifest themselves in a manner which is not consistent with discrete hard boundaries but is instead a continuous process where the value of each unit is related to that of its neighbours. Such modelling is long-established and commonplace in human geography, beginning with Geary (1954)’s discussion of issues of spatial autocorrelation, or *contagion*, when examining Irish agricultural data, and the introduction of *kriging* (Krige, 1966), where point data is used to predict values of other unknown points based on proximity as measured by distance. Metrics such as Geary’s C and Moran’s I use different approaches to quantify this phenomenon.

For aggregated areal data, a similar principle to *kriging* can lead to the construction of contiguity matrices to capture proximity not in terms of distance, but whether or not areal units are adjacent. Unlike the hierarchical framework, this process can be seen more as a moving focal point. In any particular location, it is the places immediately adjoining it which influence it the most. As the moving-window of focus shifts to the next location, it will in turn share many of the same influences but will gain some new ones. In this sense, it can be seen as capturing ‘*horizontal*’ correlations between adjoining units at the same level. When this process is extended across the study area, a different type of spatial effect is captured. In this framework, a priori groupings are irrelevant and correlation between spatial units is based only on their proximity.

#### ICAR Models

This process in areal data can be captured by conditional autoregressive (CAR) structures, first introduced by Besag (1974), of which intrinsic conditional autoregressive (ICAR) models are one type (Besag & Kooperberg, 1995). A CAR model captures spatial relationships using a contiguity matrix where all pairs of spatial units are classified as either neighbours or not neighbours. The conditional expected value for each unit then depends only on the values of adjacent units. In this way, it is an example of a Markov random field. An ICAR model assumes complete correlation between all units, the strength of which is based on their degree of contiguity. This broader dependence allows it to capture more extensive spatial relationships than the CAR structure.

#### Combination of Both Processes

But it can certainly be the case that both hierarchical and autoregressive processes are operating simultaneously.

In the hierarchical education context discussed above, a policy enacted by one education board could lead to an increase in school funding for a certain sport within their zone of governance. Such a policy would cease immediately upon crossing over into the neighbouring authority. However, the effects would not necessarily be so rigid. Should this sport prove popular, it is likely that the children will begin playing it more with their friends, regardless of what school their friends may attend. The same could then be true for friends of friends and this process would follow a contagion-like autocorrelated spatial pattern consistent with the distribution of children’s friendship groups. Thus the process can propagate in ways which are not defined by the arbitrary borders of school administration policy.

### Contribution

Our contribution is to outline an easily implementable methodology which incorporates both types of process simultaneously. It combines a hierarchical approach with a spatially autocorrelated component at the lowest level, which can not only model the process more accurately, but also allocates estimated variance according the level and type of spatial process. This aspect can be seen as analogous to an *analysis of variance* for different causes of spatial variability. Unlike a similar model applied to travel satisfaction in Beijing by Dong et al. (2016), this model is fitted within a frequentist framework, using the well-established mgcv package in R (Wood, 2011). It can be easily adapted to a range of different outcome distribution types beyond the Gaussian structure of the following example. While lme4 (Bates et al., 2015) and nlme (Pinheiro et al., 2023) are popular R packages designed specifically for multi-level modelling, they are limited in their ability to take geography into account. The mgcv package is primarily used for constructing a range of generalised additive models (GAMs), but it also contains functionality to create multilevel models equivalent to those in more specialised packages by using random effects splines. It has the further capability to combine these with Gaussian processes and Markov random fields, which are suitable for the type of spatial autocorrelation proposed above.

We demonstrate the implementation of such a model using a case study based on the UK general election of 2019.

## Data

The data used for this example are drawn from the parlitools R package (Odell, 2017), which includes a number of useful resources for analysing UK politics. It provides, among other features, convenient access to the British Election Study’s record of recent published election results, and information from the 2011 census aggregated to the constituency level.

The 2019 UK election saw a large gain in support for the Conservative Party, often at the expense of the Labour Party. Many of their seat gains occurred in constituencies which they had not recently won, despite substantial improvements over the previous two election cycles. The Labour Party, the second largest party by a substantial margin and their principal competitor, received its lowest number of MPs since 1935. This contrasts with the Conservatives gaining a majority of 80 seats, their largest since 1987. Often referred to as the collapse of Labour’s ‘*red wall*’ (Kanagasooriam & Simon, 2021), many of its losses followed a geographical pattern, notably in a collection of constituencies in the North and Midlands (Rycroft, 2020) which had been traditional Labour strongholds for many decades, albeit with declining majorities in recent elections.

In the context of UK voter behaviour, as with any data which has a geographical component, it is reasonable to hypothesise that there would be spatial processes at play in addition to differences which might be associated with socio-economic factors alone. Constituencies with similar types of census profile but in different locations do not necessarily produce similar election results. We further suspect that these spatial processes might occur in the form of both a ‘*vertical*’ hierarchical process and lowest level ‘*horizontal*’ neighbourhood effect. Dorling (2010) has described how UK society has become ever more geographically fragmented since the 1970s. If the symptoms of this were a factor in voting allegiance, we could expect a spatial hierarchical structure to pick up on this. It is also well-established that voter behaviour is often subject to a neighbourhood effect, which Pattie and Johnston (2000) summarised with the phrase “people who talk together vote together”, referencing work done by Miller (1977). The presence of such an effect would take the form of positive spatial autocorrelation among neighbouring constituencies.

The specific change in voting behaviour which we seek to model in this example is the shift in allegiance from one party to another, referred to as ‘*swing*’.

**Fig. 1:**
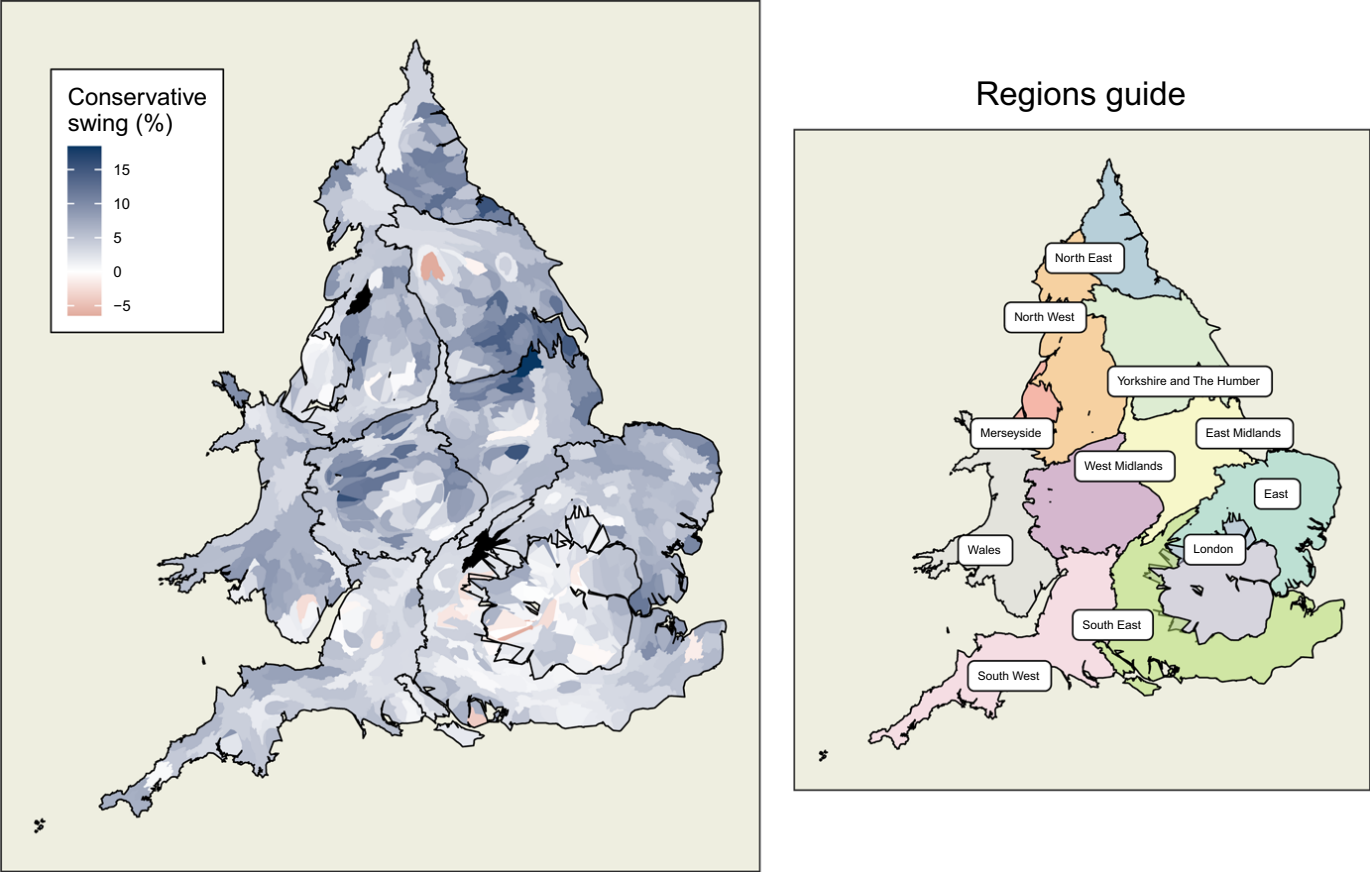
(L) Values of dependent variable, Butler swing to the Conservatives, mapped across constituencies of England and Wales. The vast majority of constituencies recorded a positive swing. Figures projected as Dougenik cartograms such that equal populations occupy equal area while maintaining constituency contiguities. (R) Guide map of the regions of England and Wales under a similar projection

### Geographical Context

Before discussing the dependent and explanatory variables of the proposed model, the geographical context is set. Data is restricted to England and Wales in this study. The reason is that, while Scotland and Northern Ireland saw interesting dynamics of their own in the 2019 election, the Conservatives and Labour were not the two primary competing parties in these parts of the UK. Neither party features to any extent in Northern Ireland, while the Scottish National Party (SNP) has dominated recent elections in Scotland.

#### Boundaries

The multilevel component of this model consists of three levels. The lowest level is composed of 571 individual *constituencies* in England and Wales.^1^ This is the level at which election results are officially reported and, due to the secrecy of the ballot, is the lowest available unit of published voting data. Each of these is nested within one of 53 *counties*. London is modelled as both a region and county. The highest level considered is the *region*, although a still higher level of *nation* has been implicitly accounted for given the exclusion of Scotland and Northern Ireland, and the classification of all of Wales as a single *region*. The *region* level has 11 components. These are Wales, Merseyside, and the nine regions of England. With the exception of Merseyside, these are essentially the NUTS level 2 administrative divisions, and coincide more or less with the former European Parliament constituencies. These regions are shown in a guide map in Fig. 1.

Merseyside has been extracted from the North West region and treated as a region in its own right for the purposes of this study. As it is well-known that Merseyside has consistently shown distinctive voting patterns in the past (Johnston et al., 2018; Kanagasooriam & Simon, 2021), particularly in respect of the Conservative party, it is here given the opportunity to show variance in its own right. Such separation has become common in recent analyses of the Brexit vote (see Gordon, 2018).

**Fig. 2:**
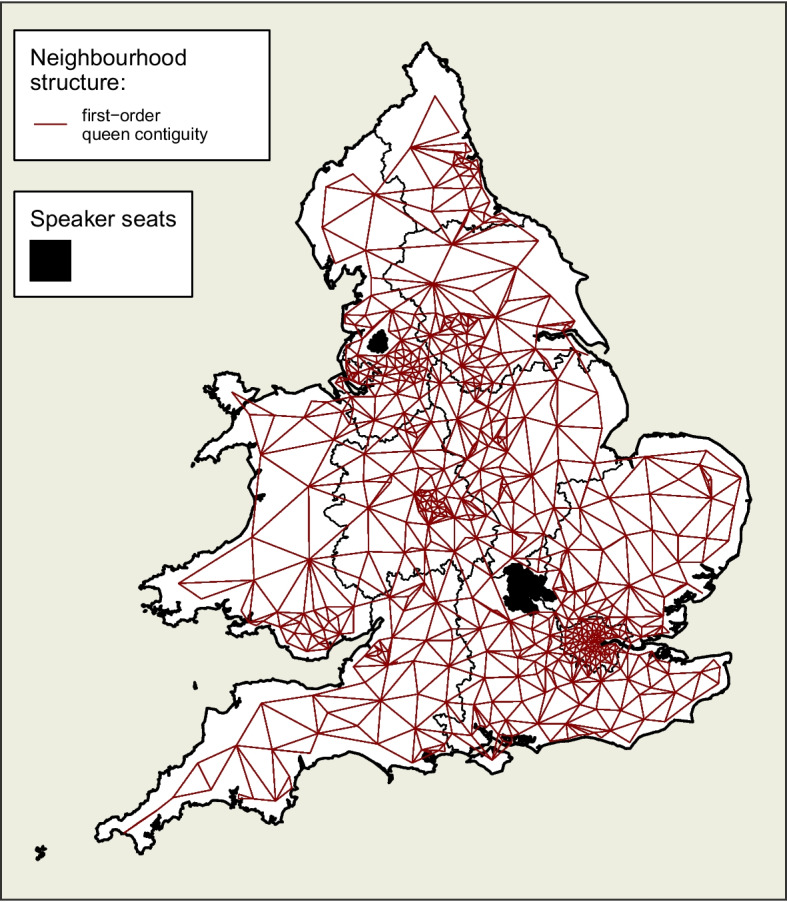
First order queen contiguity structure of constituencies in England and Wales, shown as edges radiating from nodes at the centroid of constituencies. Non-contiguous constituencies with bridge, ferry or tunnel services are also considered neighbours. Contiguity occurs regardless of region or county boundaries

#### Contiguities

The spatial autocorrelation process at the lowest (constituency) level is based on whether or not constituencies are neighbours. Here, this structure is represented by first order queen contiguity (see Fig. 2), where a constituency is considered a neighbour of another if they share at least one common point of boundary. Some additional contiguities have been added to account for invisible connectivity due to bridges and ferry crossings.

### Dependent Variable

Election swing is typically expressed as a positive or negative percentage point change. In the context of this analysis, the phenomenon under examination is the apparent change in voter preference in favour of the Conservatives and at the expense of Labour from the 2017 to the 2019 elections. The measurement of swing used here to represent this process is the ‘*conventional*’, ‘*uniform*’, or ‘*Butler*’ swing (Butler & Van Beek, 1990), which is commonly utilised in popular discourse concerning election results. It is well-known to the public as it has been used in national television coverage of election results in the UK for many decades. The Butler swing is defined as the average of the percentage point gain of party A and the percentage point loss of party B. Thus the swing to the Conservatives in the context of this election can be represented as follows:

$$ \text {Butler Swing} = \frac{(Con 2019 - Con 2017) - (Lab 2019 - Lab 2017)}{2} $$

where $Con2019$ and $Con2017$ represent the percentage of votes which were cast for the Conservative party in 2019 and 2017 respectively, while $Lab2019$ and $Lab2017$ correspond to the equivalent for the Labour party. It is calculated on the basis of total number of votes cast, including those cast for candidates other than Conservative or Labour. For example, an increase of Conservative vote share by 4.9%, combined with a decrease in Labour vote share of 7.9% would lead to a swing from Labour to the Conservatives of

$$ \frac{4.9\% - (-7.9\%)}{2}=6.4\%$$

Put another way, if the Conservatives benefited from a two-percentage point swing having initially had an equal vote share, they would now have a four-percentage point majority over Labour.

British politics has traditionally been dominated by two parties which has made this measure of swing particularly suitable. In other situations, an alternative known as the ‘Steed’ swing can be used (Curtice & Steed, 1986). This follows an identical formula except that the percentage point scores are calculated relative to the total votes received only by the two parties of interest, rather than the total number of votes cast. However, when the same two parties occupy the first two places at successive elections, as is the case in the vast majority of seats in England and Wales, the Butler swing is considered a meaningful measure of change in support (Uberoi & Baker, 2023).

**Table 1:** Explanatory variables considered by Beecham et al. (2018), separated into three thematic groupings

post-industrial / knowledge-economy	diversity / values / outcomes	metropolitan / big-city
degree educated	english-speaking	EU born, not UK
professional occupations	single-ethnicity	own home
younger adults	health not good	don’t own car
	white	private transport to work
	christian	

Such was the strength of the Conservatives’ performance in 2019 that they only experienced negative swing in 25 constituencies, almost half of which were in London. This dominance is represented by the Dougenik cartogram in Fig. 1, a style of map which is used throughout this study. It is a distorted map of England and Wales where constituency sizes are inflated or deflated, while maintaining contiguities, such that equal population is represented by equal space on the map (Dougenik et al. 1985, implemented by the cartogram R package by Jeworutzki 2020). Such a map is particularly informative in this case because most small constituencies are very densely populated and vice versa. A heavily populated part of London would be virtually invisible on a standard choropleth map projection. These maps overcome this problem in that every unit area contains exactly the same number of people.

### Explanatory Variables

The explanatory variables chosen for this study come from the 2011 census, the most recent prior to the election. They are based on those proposed by Beecham et al. (2018) in their examination of spatial variation of voter behaviour in the 2016 Brexit referendum. They considered covariates based on “the media discourse around the Leave vote: that of the ‘*left-behind*’ and of the varying experiences of de-industrialisation” (Cox, 2016). This is consistent with the aforementioned spatial fragmentation process (Dorling, 2010). Places described as ‘*left-behind*’ are often characterised by “chronic low skills, socially conservative and nativist values”, as opposed to other areas with “more affluent, highly-educated and diverse populations” (Goodwin & Heath, 2016). The candidate variables and their thematic groupings considered by Beecham et al. are reproduced in Table 1.

Preliminary models showed that those variables in the *metropolitan / “big-city”* category were not significant in the context of swing in the 2019 election. For this reason, the have been omitted from this study.

**Table 2:** Reduced list of relevant explanatory variables grouped by justification, and the choice of one representative variable from each group for subsequent models

grouped candidate variables	justification / theory	representative variable
1		
degree educated	*post-industrial / knowledge-economy /*	degree educated
professional occupations	*peripherality*	
2		
younger adults	*life outcomes /*	health not good
health not good	*young people*	
3		
english-speaking	*ethnic / cultural diversity /*	white
single-ethnicity	*values*	
white		
christian

**Table 3:** Description of explanatory variables used in subsequent models

explanatory variable	calculation from census
degree educated	*percentage of population with level 4 qualification or higher*
health not good	*percentage of the population self-reporting ‘poor’, ‘bad’, or ‘very bad’ health*
white	*percentage of population of white ethnicity*

**Fig. 3:**
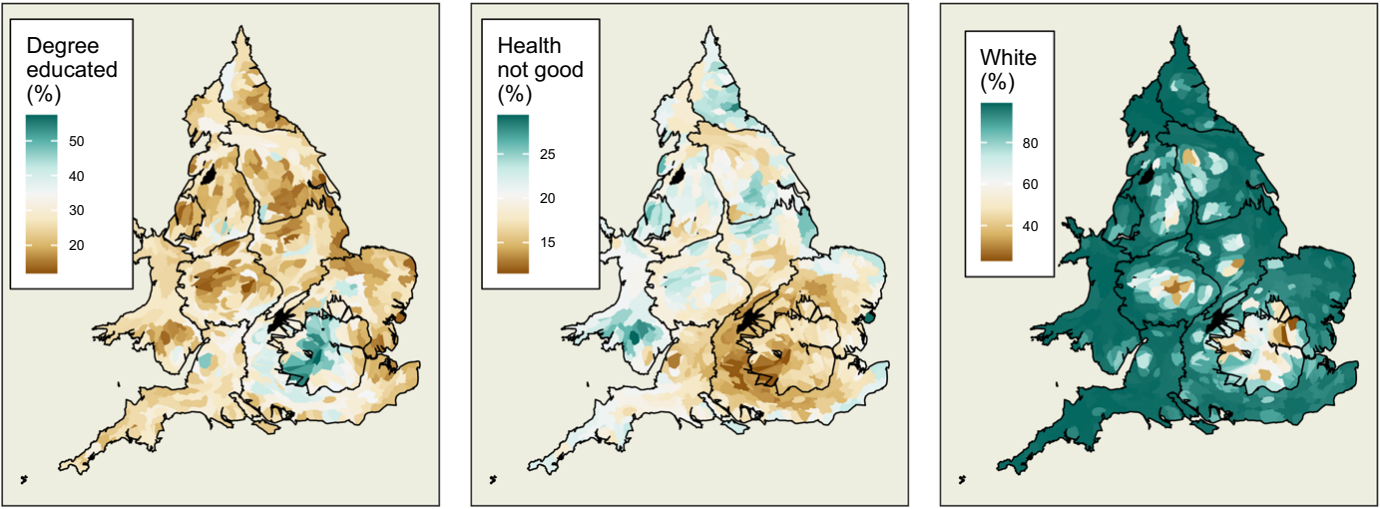
Values of independent variables mapped across England and Wales. Figures projected as Dougenik cartograms such that equal populations occupy equal area while maintaining constituency contiguities

Upon examination of the remaining variables, shown in Table 2, three groups can be discerned. Each contains highly correlated variables which also show similar associations with swing. In order to mitigate against multicollinearity, one variable was chosen from each of these groups for this model as representative of this category. Table 3 shows how these three explanatory variables are calculated.

The variable *degree educated* is the percentage of the population of a constituency with at least a level 4 qualification (such as undergraduate degrees or similar qualifications). Figure 3 shows that the highest levels of this measure are concentrated overwhelmingly in London and its environs. Small pockets of high values can also be seen in other core cities such as Manchester and Bristol. The lower scores are found in areas which might be considered more peripheral, in particular a strip from South Wales to the Humber estuary.

The variable *health not good* is the percentage of the population in the census who self-report their health as ‘*poor*’, ‘*bad*’, or ‘*very bad*’. It is assumed that there is a strong association between health outcomes and overall quality of life. It is also biologically more likely that areas with a higher proportion of younger people will have lower levels of poor health, other things being equal. Looking again at Fig. 3, this variable shows the strongest indication of a north-south divide in England and Wales. It suggests a stark difference between values either side of a line drawn from the Bristol Channel to the Lincolnshire coast. Areas with particularly poor health outcomes can be seen in parts of South Wales, Merseyside and the North East. Unlike many of the other variables, London does not score at either extreme of *health not good*. Instead, it is areas to the West of London, stretching across to Bristol which show the lowest levels of poor health.

The final independent variable is *white*. This is the percentage of a constituency’s population who identify as being of exclusively white ethnicity. Figure 3 shows that this measure of ethnic diversity has a different pattern again. While the areas below 50% white are predominantly urban constituencies, it is notable that not all large cities fall into this category. Some cities are composed of a much more ethnically diverse population than others.

Prior to constructing models, these explanatory variables are scaled such that they exhibit mean of zero and standard deviation of one. This means that units of increase in a dependent variable refer to unit changes in standard deviation of percentage points of that variable, which makes comparability of effects more interpretable. Furthermore, the mean of zero allows intercepts in regression models to be viewed as the estimated level of a dependent variable with all explanatory variables held at their mean value.

**Fig. 4:**
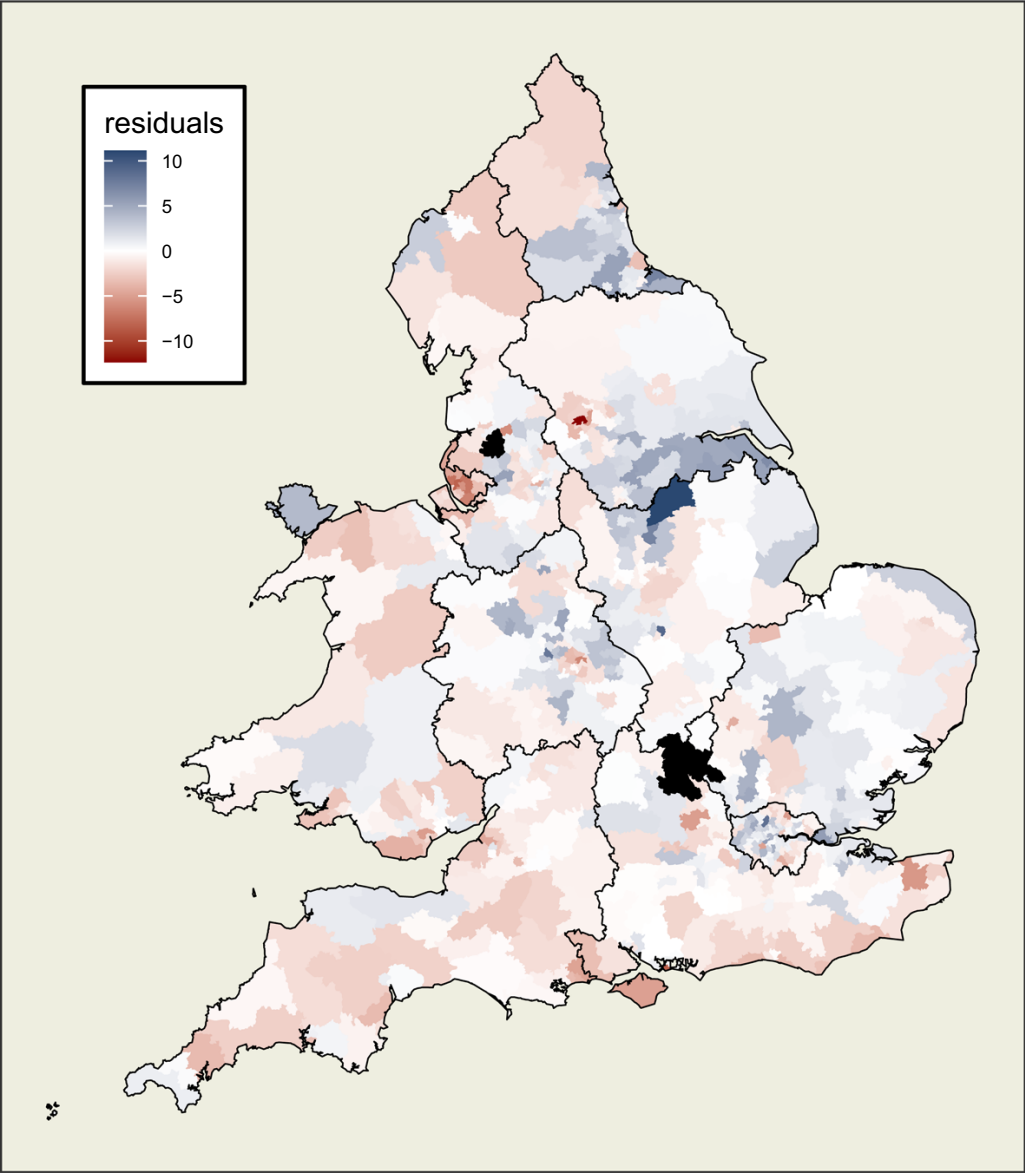
Map of residuals from a simple linear model which does not take geography into account. Regions such as the South West and Merseyside appear to be almost completely red (overprediction of swing), the North East show a block of red alongside a block of blue, while a blue pattern of underprediction spreads across the boundary between the East Midlands and Yorkshire and the Humber

## Model

A simple linear regression using these three explanatory variables and no spatial component, that is without hierarchy or spatially autocorrelated effects, produces residuals as shown in Fig. 4. It is clear that swing in certain regions is predominantly over or under-estimated. These can be observed as block patterns of red or blue respectively. For example, the South West are Merseyside are overwhelmingly ‘red’ indicating that Labour actually performed better than would be predicted by this model. It also suggests lower level county variations in places such as the North East where there are major blocks of both ‘red’ and ‘blue’, again indicative of systemic error in one direction or another. All of this is suggestive of the presence of unaccounted-for hierarchical spatial processes. There are also clusters of similarly coloured constituencies which cross over regional boundaries, most notably between Yorkshire and the Humber and the East Midlands. These suggest autocorrelated processes, operating beyond a hierarchical structure, which have not been captured.

Our proposed modelling framework allows for the inclusion of both hierarchical and autocorrelated effects, both of which are suspected to be present from a theoretical perspective and from examination of the residuals in Fig. 4. The model described below, a hierarchical model with a spatially autocorrelated random component for each constituency, is calibrated to test for the presence of such processes and estimate them at different scales.

### Hierarchical Component

The form of the hierarchical model is as follows: Butler swing is taken as the response variable, and is assumed to have a linear relationship with each explanatory variable, controlling for the others. The three explanatory variables are *degree educated*, *health not good*, and *white*, as discussed earlier. The level of swing for any individual constituency is modelled as an intercept plus a linear combination of these three covariates. Each intercept and slope coefficient is composed of the overall mean slope and intercepts for England and Wales, plus a differential of intercept and slopes for each region relative to the overall means, and another differential for each county relative to the means of the region in which it is nested.

The hierarchical structure means that, for example, a county intercept of zero would indicate that the county’s mean is no different than the mean of the region in which it lies, controlling for the independent variables. Similarly, a regional level slope coefficient of zero for a particular explanatory variable would indicate that the association of that variable at the regional level is no different than the overall mean coefficient level, again holding other components constant.

In mixed models, the random intercepts and coefficients are not modelled directly. Instead, they are assumed to be normally distributed with mean zero and their variance and covariances are estimated using, in this case, restricted maximum likelihood (REML). By examining whether confidence intervals around these variances include zero, hypotheses can be tested as to whether the slopes and coefficients vary significantly relative to the higher administrative level. The ‘*best linear unbiased predictors*’ (BLUPs) of slope and intercept for individual regions and counties can then be calculated from the estimated variance components using the empirical Bayes method, which involves computing the posterior distribution of the random effects at each level of the hierarchy and taking the conditional mean as the BLUP for each unit at that level.

### ICAR Component

In addition to this, a spatially smoothing intrinsic conditional autoregressive (ICAR) component, as discussed earlier, is added at the lowest level to account for spatial dependence between adjacent constituencies which is not captured by the nested structure. The degree to which each neighbour of a given constituency influences it is proportional to the total number of neighbours of that constituency. Neighbours are defined by adjacency in this example but it could equally represent degree of connectivity by infrastructure, patterns of commuting, location of population concentrations, etc. A constituency is not considered to be its own neighbour, and each constituency is considered to be a neighbour of another if their boundaries share at least one common point. In the case of our model, the set of values generated by this component can be seen as random effects for each individual constituency.

Finally, there is an error term for each constituency which has mean zero and variance $\sigma ^2$. This term accounts for differences in swing which can not be attributed to the three chosen independent variables, their nested location within county and region, or the constituency neighbourhood structure.

### Model Structure

The structure of the model is outlined below:

$$\begin{aligned} y_{ijk}= & {} \beta _0 + \beta _1 degree_{ijk} + \beta _2 health_{ijk} + \beta _3white_{ijk}\nonumber \\{} & {} + b_{0i} + b_{1i} degree_{ijk} + b_{2i} health_{ijk} + b_{3i}white_{ijk\nonumber }\\{} & {} + b_{0ij} + b_{1ij} degree_{ijk} + b_{2ij} health_{ijk} + b_{3ij}white_{ijk}\nonumber \\{} & {} + \gamma _{l}|\gamma _{m}, l\ne {m}\\{} & {} + \epsilon _{ijk} \end{aligned}$$

where $y_{ijk}$ is the swing in constituency $k$ in county $j$ in region $i$ for

- $$i = 1, ... , 11$$ regions,
- $j = 1,..., J_{i}$ counties within region $i$,
- $k = 1,..., K_{ij}$ constituencies within county $j$ within region $i$, and
- $$l = 1,..., 571$$ individual constituencies.
- $\beta _0$, $\beta _1$, $\beta _2$, $\beta _3$ are fixed effects.
- $b_{0i}$, $b_{1i}$, $b_{2i}$, $b_{3i}$ are the random effects (intercept and three slopes) associated with region $i$,
- $b_{0ij}$, $b_{1ij}$, $b_{2ij}$, $b_{3ij}$ are the random effects (intercept and three slopes) associated with county $j$ in region $i$.
- $\epsilon _{ijk}$ are independent normally distributed error terms.

Rather than estimate each of the random effect coefficients directly, the variance of each random effect is instead estimated. For the region and county level random effects, each is assumed to be independent of the others within its level, and to be normally distributed with mean of zero. This independence is a key restriction in multi-level modelling with mgcv as opposed to other packages.

The $\gamma _{l}$’s are constituency level random effects which model the spatial interactions at the lowest level of the model, based on an ICAR distribution. Let there be $m = 1,..., M$ potential neighbouring constituencies, where $M=L=571$. Each $\gamma _{l}$ is conditional on the sum of the weighted values of its neighbouring $\gamma _{m}$’s ($\text {w}_{lm}\gamma _m$) and has unknown variance. As a constituency is not a neighbour to itself, the full conditional distribution can be written as follows:

$$ \gamma _l | \gamma _m,l\ne {m} \sim \mathcal {N}\bigg (\frac{\sum _{l\ne {m}}{\gamma _l}}{d_l},\frac{\sigma _{l}^2}{d_l}\bigg ) $$

where the term $d_l$ represents the number of neighbours. Thus the mean of each $\gamma _l$ is equal to the average of its neighbours, while its variance decreases as the number of neighbours increases.

The joint specification of the ICAR random vector $\gamma$ when centred at $0$ with common variance $1$ rewrites to the pairwise difference formulation:

$$ \gamma \propto \text {exp}\bigg (-\frac{1}{2}\Sigma _{l\ne {m}}(\gamma _l-\gamma _m)^2\bigg ) $$

To overcome the problem of unidentifiability, the constraint $\Sigma _L\gamma _l=0$ is added to centre the model.

## Results

The aim of this modelling structure was to enable us

1. to test for the presence of spatial effects resulting in different associations between covariates and the dependent variable according to geography, taking into account both hierarchical and autoregressive spatial processes (*spatial heterogeneity*),
2. and also to estimate the relative variance associated with each type of process at different spatial scales (‘*analysis of variance*’ of spatial processes).

**Fig. 5:**
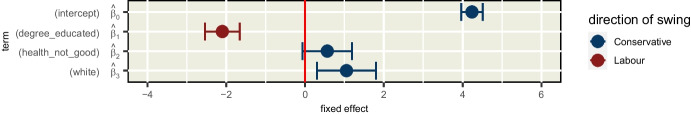
Plot of fixed or global intercept and coefficients from combined model, coloured according to direction of association with swing. A higher proportion of people of white ethnicity in an average constituency is associated with a swing to the Conservatives while the opposite is true for increases in the proportion of degree-educated

Firstly, the global intercept and coefficients, having controlled for region, county and constituency spatial effects, are shown in Fig. 5. They suggest that on average, an increase in the *white* proportion of a constituency by one standard deviation, which can be interpreted as lower diversity, is significantly associated with a 1 percentage point swing to the Conservatives. In contrast, an increase of similar size in *degree educated* people among a constituency’s population is associated with a 2.1 percentage point swing away from them. However, these mean global effects do not tell the full story.

### Region Level

**Fig. 6:**
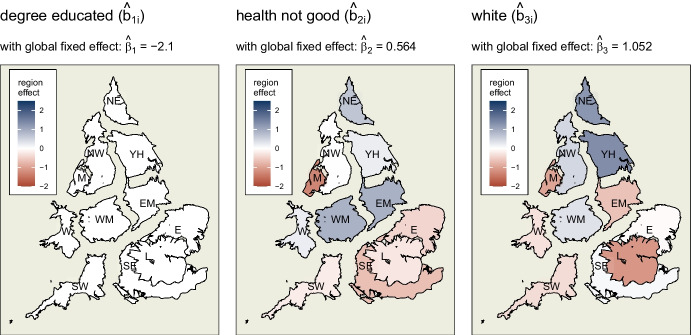
Regions of England and Wales, coloured according to direction and magnitude of region-level random effects of each covariate with swing to the Conservatives. The global fixed effect from which these divergences occur is shown above each map. Unlike ‘health not good’ and ‘white’, ‘degree educated’ does not show significant divergence at this level from its global coefficient

Looking at the random effects at the region level in Fig. 6, it is clear that divergences in association occur. This is not the case, however, for variable 1, *degree educated*. At this level, its association with swing does not vary significantly from the global mean ($\hat{\beta }_1$) of $-2.1$. An increase in the percentage of *degree educated* voters within a constituency is associated with a swing away from the Conservatives.

Looking at the various random coefficients for *health not good* ($\hat{\beta }_{2i}$’s for $i$ regions), not only do they operate in different directions, the negative values are often sufficiently large to counteract the positive global effect ($\hat{\beta }_2$) of $0.564$. Thus, while a higher proportion of a constituency’s population with poor health is generally associated with an increase in swing to the Conservatives, this effect is much stronger in the Midlands regions and the North East, but is reversed in Merseyside, and neutralised in the East and the South East.

A similar process occurs for the third covariate, although the heterogeneities occur in different regions. The global association of a higher proportion of *white* ethnicity in a constituency and swing to the Conservatives ($\hat{\beta }_3$) is positive. This is much more strongly the case in the North East and Yorkshire and the Humber. However, in the East Midlands, London and Merseyside, the counter-acting negative random effect is enough to reverse the direction of association. It is also drawn closer to zero for Wales and the South West.

Table 4 shows that these region-level hierarchical effects account for 18.5% of total variance in the data.

**Table 4:** Variance explained by model, with associated measures of significance, at different levels and for different spatial processes

Level	Variance	Variance %	Cumulative variance %	F test p-val	
Region					
$\sigma ^{2}_{region,int}$	<0.01	<0.01	<0.01	0.074	.
$\sigma ^{2}_{region,degree}$	<0.01	<0.01	<0.01	0.344	
$\sigma ^{2}_{region,health}$	0.63	7.2	7.2	0	***
$\sigma ^{2}_{region,white}$	0.99	11.2	18.5	0.001	***
County					
$\sigma ^{2}_{county,int}$	<0.01	<0.01	18.5	0.082	.
$\sigma ^{2}_{county,degree}$	0.44	5	23.5	0	***
$\sigma ^{2}_{county,health}$	<0.01	<0.01	23.5	0.54	
$\sigma ^{2}_{county,white}$	0.34	3.9	27.4	0.045	*
Constituency (ICAR)					
$\sigma ^{2}_{constituency,ICAR}$	3.63	41.3	68.7	0	***
Residuals					
$\sigma ^{2}$	2.74	31.3	100		***

There is significant variation in the association with ‘health’ and ‘white’ at region level, while at county level ‘degree’ and ‘white’ show significant divergence

Signif. codes: 0 ‘***’ 0.001 ‘**’ 0.01 ‘*’ 0.05 ‘.’ 0.1 ‘ ’ 1

### County Level

**Fig. 7:**
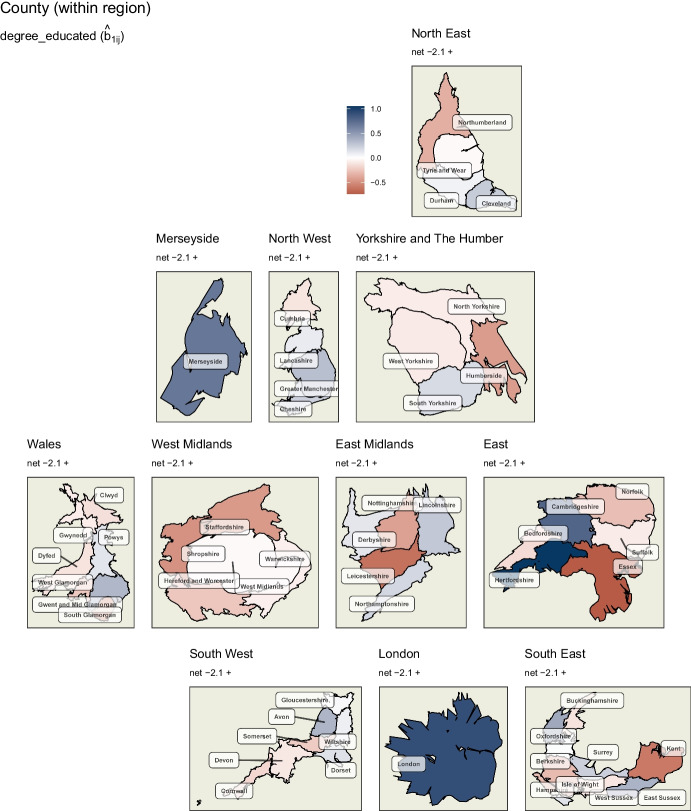
Random effects of ‘degree educated’ variable at the county level of England and Wales. Particularly strong within-county variation can be observed in the East

Looking at the county level random effects of the first covariate, *degree educated* (Fig. 7), we can see within-region deviations. These are particularly pronounced in the East. However, while they do strengthen or weaken the negative association (-2.1) of this covariate with swing to the Conservatives across all regions, they are not sufficient at any location to alter its direction.

Table 4 shows that there is no further significant random effect divergence at county level for the second variable, *health not good*.

**Fig. 8:**
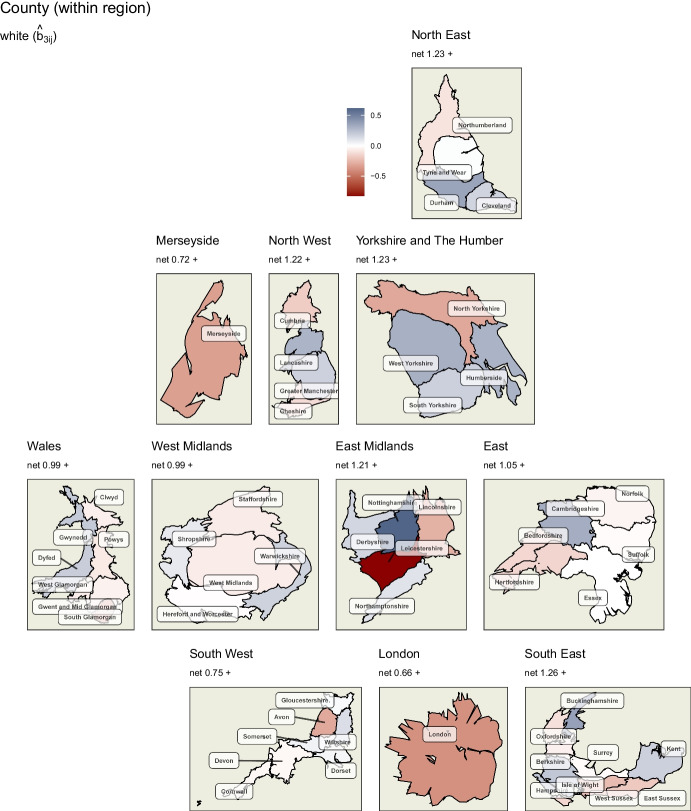
Random effects of ‘white’ variable at the county level of England and Wales. Particularly strong within-county variation can be observed in the East Midlands

Similarly to *degree educated*, the *white* covariate (Fig. 8) shows strong within-region county level variation, particularly in Nottinghamshire and Leicestershire in the East Midlands, which show a strengthening and a reversal respectively of the positive association between low ethnic diversity and swing to the Conservatives within this region.

Referring again to Table 4, these county-level hierarchical effects account for 8.9% of total variance in the data and are much less impactful than those at the region level.

### Net Hierarchical Effects

**Fig. 9:**
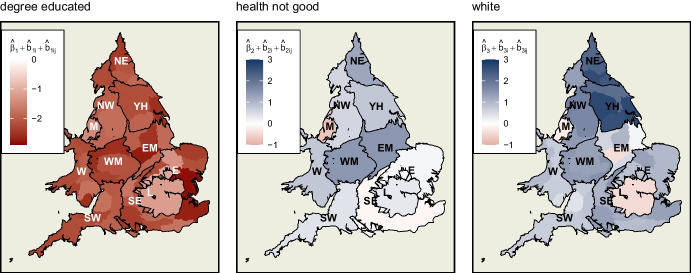
Net hierarchical effects of each variable. These are the sum, for each coefficient, of its fixed effect and two random effects (at region and county level), showing the spatial heterogeneity accounted for by the hierarchical component of the model. ‘Degree educated’ is negatively associated with swing to the Conservatives across England and Wales, albeit to different extents. ‘Health not good’ and ‘white’ show not only different magnitudes but also different directions of association with swing in different regions and counties across the study area

These hierarchical random effects at region and county level can be added to the global coefficients to show a picture of the net associations and their variability across England and Wales (Fig. 9). In the case of *degree educated*, for instance, this is the sum of the global coefficient ($\hat{\beta }_1$) and the random effects for region ($\hat{b}_{1i}$) and county ($\hat{b}_{1ij}$).

As discussed above, the level of *degree educated* in a constituency is negatively associated with swing to the Conservatives in all locations, but less so in some counties. Looking at *health not good*, higher levels of poor health outcomes within a constituency are associated with a swing to the Conservatives to varying degrees, except for Merseyside and the East and South East where it is reversed or negligible. Finally, the *white* covariate suggests that lower levels of ethnic diversity in a constituency are associated with swing to the Conservatives in most of England and Wales. This is particularly strong in certain counties of the North East and Yorkshire and the Humber. However, the reverse is also observed in London, Merseyside and part of the East Midlands.

### Constituency Level

The final component of the model is the set of spatially autoregressive terms which, according to Table 4, account for 41.3% of the variance. They can be interpreted as random constituency-specific effects which account for further differences in voting behaviour which are not associated with the chosen census variables, nor with location within a region or county. Instead, they exhibit a pattern consistent with the defined neighbourhood contiguity structure and the resultant expected diffusion of political attitudes across nearby constituencies.

**Fig. 10:**
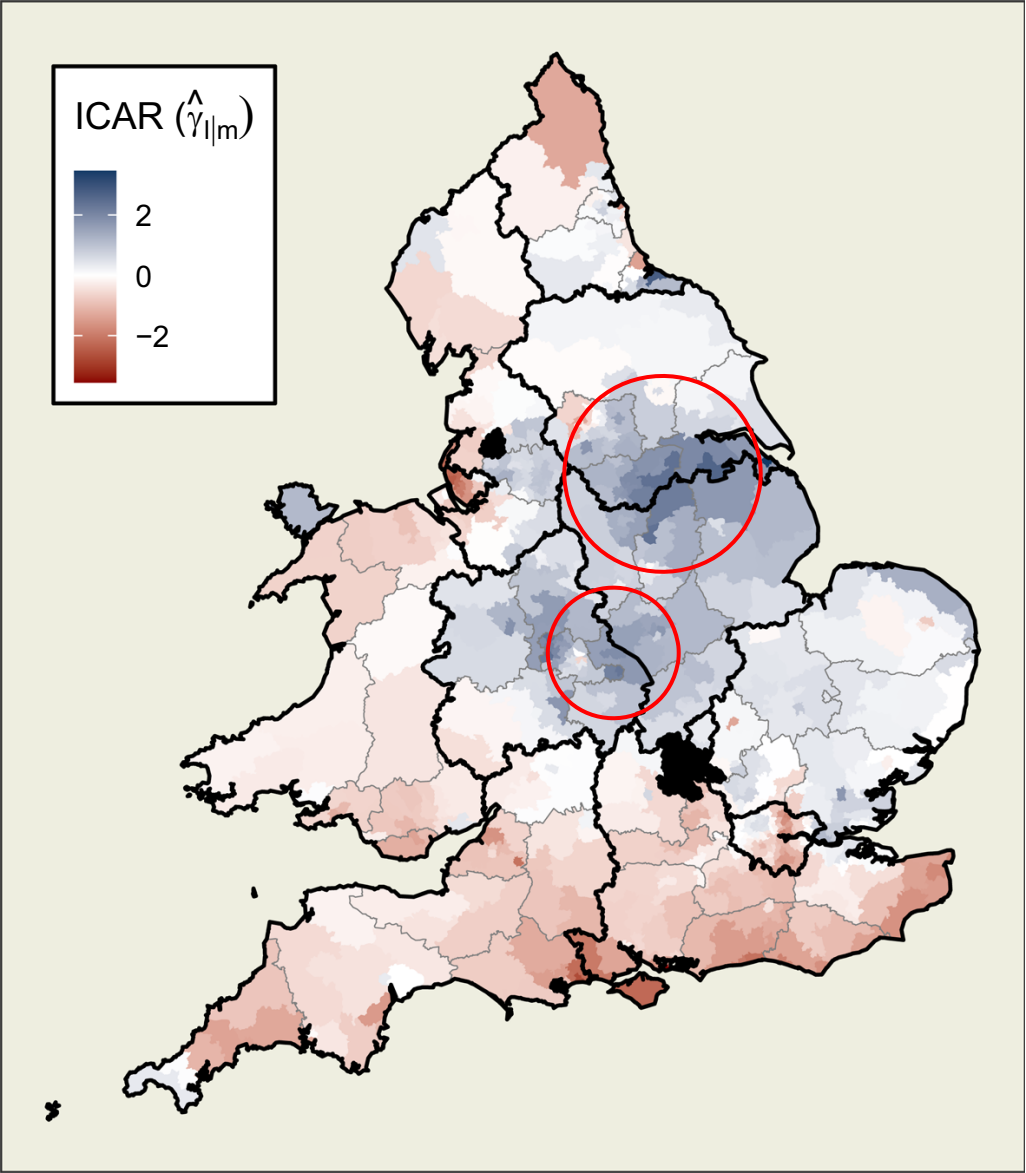
The spatially autoregressive (in this case, ICAR) component at the lowest level (constituency) of the model. It shows a blue area of increased tendency to swing to the Conservative party, surrounded by a paler band, and red areas to the south, west and parts of the north where the tendency is to swing to Labour, controlling for the covariates and hierarchical effects. Areas of clear cross-regional spillover of effects are highlighted with red circles

As can be seen in Fig. 10, the pattern of the outcome does not align with the regions and county boundaries. Instead, we see a large and generally blue central area which is surrounded by a paler white section. This blue area, lying within parts of the East and West Midlands and Yorkshire and the Humberside, displays an increased tendency to swing to the Conservatives in 2019, over and above what the census variables and hierarchy would predict. There are patterns of particularly strong blue within this area which cross directly over regional boundaries (as highlighted by the circled areas of the map).

Conversely, across southern England, through Wales, and into parts of the far-north, the predominant trend is a lower level of swing to the Conservatives than would otherwise be predicted.

While the model does not specify what the driving forces for these divergences above and below the expected levels of swing might be, it does detect their presence in the form of a hypothesised pattern of spatial diffusion. Such an insight could be useful to political scientists. It is, for example, consistent with how a set of shared attitudes or political culture in one part of the country would change gradually across neighbours. Similarly, support for a party due to local policy proposals, the benefits or drawbacks of which would not be restricted to that location alone, could be expected to show similar autoregressive patterns.

### Alternative Autoregressive Components

The model presented above features a multilevel structure with an autocorrelated random effect at the lowest level for each constituency. This framework, however, also allows for more complex structures than this. For example, in addition to the random intercepts and slopes provided for in the hierarchical component, we have the option of using either

1. a spatially autocorrelated random intercept at constituency level (as we did in this model),
2. spatially autocorrelated random slopes for each covariate in each constituency, or
3. both together.

To decide which of these three options was most suitable for this particular dataset, their performances can be compared. The fitting of such spatial models using the mgcv package requires the tuning of a parameter *k* which is the number of basis functions used to generate the autoregressive smoothing. Lower values of *k* lead to a smoother result. This is because *k* represents the number of components from the eigen decomposition of the variance-covariance structure which are to be used. Not all can be used because there are not enough data points for this to be computable. The *k* value has been optimised for each model such that the Akaike information criterion (AIC) is minimised, striking a balance between goodness of fit and model complexity.

Shown in Table 5 are performance metrics for each of these model combinations, named models **1-3**. Of these three potential structures, model **1**, which we have been discussing, has the best performance metrics and was deemed the most suitable structure for modelling this particular dataset. Such a process can be used to find the most suitable structure for any potential dataset.

**Table 5:** Performances of a hierarchical structure with different combinations of spatial autocorrelation processes at lowest constituency level

Model	Autoregressive spatial process(es)	AIC	RMSE	adjR2	Loglik
1	constituency component	2336	1.43	0.76	−1015
2	varying coefficients	2373	1.62	0.73	−1086
3	constituency component + varying coefficients	2381	1.64	0.73	−1094

Models 1 and 2 each incorporate one type of process while Model 3 includes both. Model 1 performs best on all metrics

**Fig. 11:**
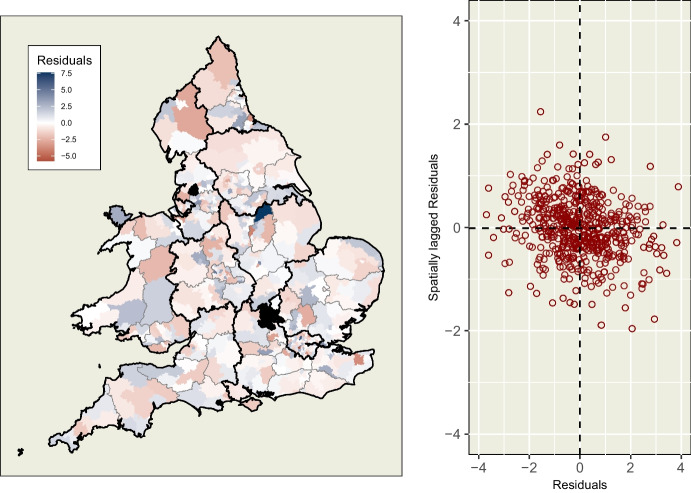
(L) Residuals from model, mapped by location, which appear by visual inspection to be randomly distributed, and (R) dot-plot of residuals of constituencies against their spatially lagged neighbours which shows neither a positive nor negative association between a constituency’s residuals and those of its neighbours

### Spatial Diagnostics of Model

Finally, unlike the pattern previously observed in Fig. 4, the residuals from our model, mapped in Fig. 11, show no evidence of any remaining unaccounted-for spatial processes. The spread of positive and negative residuals across the study area appear random by visual inspection, and a Moran’s I test of randomness supports this observation.

## Discussion and Conclusion

This study presents a framework for analysing spatial data which takes account of the different ways in which these spatial processes may be likely to occur. It simultaneously incorporates a ‘*vertical*’ set of relationships between nested geographical areas with certain hypothesised shared characteristics, and a ‘*horizontal*’ covariance among the lowest level units according to contiguity.

In the context of analysing elections, the model structure described above not only succeeds in modelling voting behaviour more accurately than less complex models, but also provides insight into the processes generating the results. It supports the hypothesis of different patterns of association of socio-demographic profile with voting behaviour in different parts of the study area.

It also identifies patterns of places which are more or less likely to swing to the Conservatives for reasons which can not be attributed to the census explanatory variables or hierarchical location, but which are consistent with a *neighbourhood* effect. These patterns are not immediately obvious from the raw data or from models which do not account for location in this way. Insights such as these could be valuable for political theorists.

The framework allows for the testing of different combinations of autocorrelation structure to find which types of spatial processes are most appropriate in a given context. Here, we chose what was essentially an autocorrelated random intercept for each constituency, but we could also fit a set of random slopes if the data suggested that such a structure was more appropriate. The model is fitted in a frequentist restricted maximum likelihood framework using the well-established mgcv package.

Another feature is the ability to estimate the relative contribution of each spatial process to overall variation. In this example, it apportions about 27% of variance to hierarchical processes, two-thirds of which occur at the region level, and a further 41% to *spillover* effects at the lowest constituency level. Furthermore, the spatial heterogeneity of the three covariates is shown to operate at different levels within the hierarchy. The variance in association of swing with *degree educated* occurs at the smaller-scale county level, while that of *health not good* and *white* is stronger at the region scale. Such patterns, which again are not otherwise immediately apparent, can contribute to the research of political scientists who are interested in understanding the geography of voting patterns.

## Data Availability

The code and data used to create these results can be found at https://github.com/horankev/swing_project.

## Footnotes

1 There are actually 573 constituencies in England and Wales but two have been excluded as they were held by the respective Speakers of the house in 2017 and 2019, and are traditionally not contested. In all constituency-level maps in this paper, these seats are coloured black.
